# Trend of avoidable mortality due to circulatory system diseases and ischemic heart diseases in Rio Grande do Sul (2000–2023): an ecological analysis

**DOI:** 10.1590/1806-9282.20250627

**Published:** 2026-03-30

**Authors:** Franciele Souza Santos, Murilo Santos de Carvalho, Juvenal Soares Dias da Costa

**Affiliations:** 1 Universidade do Vale do Rio Dos Sinos, Postgraduate Program in Public Health – São Leopoldo (RS), Brazil..; 2 Universidade Federal de Ciências da Saúde de Porto Alegre, Postgraduate Program in Rehabilitation Sciences – Porto Alegre (RS), Brazil.

**Keywords:** Cardiovascular diseases, Myocardial ischemia, Mortality, Time series analysis, Public health

## Abstract

**OBJECTIVE:**

The aim of this study was to analyze trends in avoidable mortality due to circulatory system diseases and ischemic heart diseases among individuals aged 30–69 years across the health macro-regions of Rio Grande do Sul, Brazil, from 2000 to 2023.

**METHODS:**

An ecological time-series study was conducted using data from the Mortality Information System and Department of Informatics of the Unified Health System, with calculation of age- and sex-adjusted mortality coefficients.

**RESULTS:**

A reduction in mortality from circulatory system diseases and ischemic heart diseases was observed among women in almost all macroregions, except for the Central-West. Among men, decreasing trends in mortality from circulatory system diseases were identified, except in the Vales macro-region and in the state overall. Mortality was higher among men and increased with age, with the highest coefficients observed in the South, Central-West, and Metropolitan macro-regions.

**CONCLUSION:**

A downward trend in mortality was observed despite population aging, which may be attributed to health policies that strengthened the Unified Health System, including the expansion of the Family Health Strategy, the Hypertension and Diabetes Mellitus Care Reorganization Plan, and the Popular Pharmacy Program, as well as measures for chronic disease control and health promotion.

## INTRODUCTION

Avoidable mortality refers to deaths that should not occur with appropriate and timely treatment, meaning they are preventable and serve as an indicator of the effectiveness of healthcare services or systems^1^.

The Brazilian Ministry of Health, through a consensus of experts, developed lists of avoidable mortality for children under 5 years old and individuals aged 5–75 years^2^. In the present study, the population aged 5–69 years was considered, following the same age limit as the Sustainable Development Goals for noncommunicable diseases^3^.

According to the Global Burden of Disease, circulatory system diseases remain among the leading causes of death worldwide^4^. Although mortality from circulatory system diseases has declined in many regions, the number of deaths remains high in most low-and middle-income countries^5^. Among circulatory system diseases, ischemic heart diseases are predominant^6,7^. In Brazil, circulatory system diseases are also among the leading causes of death, with ischemic heart diseases being particularly significant^8,9^.

A systematic analysis of mortality rates allows for comparisons between different locations and populations, facilitating a better understanding of disease patterns and enabling the development of more precise health policies and interventions^4,10^.

Thus, the objective of this study was to analyze the trend of avoidable mortality due to circulatory system diseases and ischemic heart diseases among individuals aged 30–69 years residing in the health macroregions of Rio Grande do Sul (RS), Brazil, from 2000 to 2023.

## METHODS

This is an ecological time-series study that examined the trend in avoidable mortality from circulatory system diseases and ischemic heart diseases among individuals aged 30–69 years residing in the health macro-regions of Rio Grande do Sul (RS), Brazil, from 2000 to 2023.

RS is the southernmost Brazilian state, with an area of approximately 282,000 km^2^ and a population of over 11 million inhabitants distributed across 497 municipalities. The health system is organized into seven health macro-regions: Metropolitan, Missions, North, South, Serra, Valleys, and Central-West. Each macro-region encompasses several health regions, which in turn are composed of municipalities.

Mortality data were obtained from the Mortality Information System, based on the chapter of circulatory system diseases included in the Brazilian List of Avoidable Causes of Death: Hypertensive disease, except secondary hypertension (International Classification of Diseases [ICD]-10: I10–I13); Ischemic heart disease (ICD-10: I20–I25); Atherosclerosis (ICD-10: I70); Heart failure (ICD-10: I50); and Cerebrovascular diseases (ICD-10: I60–I69). Data were compiled by sex (male, female, and total) and age group (30–39, 40–49, 50–59, and 60–69 years), according to place of residence and year.

Population data were obtained from the Brazilian Institute of Geography and Statistics and were available through the Department of Informatics of the Unified Health System (DATASUS), via the TabNet platform, under the “Demographic and Socioeconomic” category. These data provided population estimates by macro-region, age group, and sex for the period 2000–2023.

Mortality coefficients were calculated in Microsoft Excel using the formula: [(annual number of deaths by sex and age group/resident population by sex and age group per year)×10,000 inhabitants]. The coefficients were age-adjusted using the direct method, with the 2010 RS population as the standard, maintaining the same age groups to eliminate differences in age structure across macro-regions.

Trend analysis of the standardized mortality coefficients was conducted by sex in each macro-region and in the state overall, and by sex and age group within the state. The trend analysis was performed using Prais-Winsten^11^ generalized linear regression. The regression results were expressed as annual percent change, indicating increasing, decreasing, or stable trends, along with corresponding 95%CIs and statistical test results.

Studies using secondary data from publicly available information systems are exempt from evaluation by Research Ethics Committees, in accordance with Resolution No. 466/2012 of the National Research Ethics Commission.

## RESULTS

A total of 190,175 avoidable deaths from circulatory system diseases were recorded in Rio Grande do Sul (RS) among individuals aged 30–69 years between 2000 and 2023. Mortality was higher among men, and a direct relationship was observed between mortality coefficients and age groups across all macro-regions. The highest mortality coefficients were found in the South, Metropolitan, and Central-West macro-regions (Table 1).

**Table 1:** Distribution of standardized coefficients of avoidable mortality due to circulatory system diseases by sex, across health macro-regions and in the state of Rio Grande do Sul, 2000–2023.

Year	Vales			Metropolitan			North			serra			Missioneira			South			Central West			RS		
	M	FEM	Total	M	FEM	Total	M	FEM	Total	M	FEM	Total	M	FEM	Total	M	FEM	Total	M	FEM	Total	M	FEM	Total
2000	30.2	15.9	22.9	34.0	19.1	26.0	23.0	13.2	18.0	22.3	15.0	18.6	23.2	16.8	19.9	33.7	18.9	26.0	31.0	18.2	24.4	16.0	9.8	12.8
2001	25.7	15.9	20.7	29.8	17.5	23.2	21.6	12.9	17.2	21.7	13.5	17.5	26.2	13.8	19.9	32.4	17.2	24.5	25.7	17.1	21.3	14.6	9.0	11.7
2002	26.4	13.3	19.7	28.1	16.4	21.9	20.8	11.8	16.2	18.3	10.6	14.3	23.0	14.4	18.6	28.9	16.2	22.3	26.2	14.7	20.3	13.7	8.2	10.9
2003	23.8	14.0	18.8	27.0	15.2	20.7	20.9	11.9	16.3	17.2	10.7	13.9	22.5	11.5	17.0	20.0	11.8	15.9	24.6	16.5	20.4	13.1	7.9	10.5
2004	22.3	14.6	18.4	25.3	14.1	19.3	19.1	13.2	16.1	20.6	11.3	15.8	42.3	25.3	17.2	27.4	17.9	22.5	28.6	13.8	21.0	12.9	7.8	10.3
2005	22.0	12.2	17.0	23.9	13.6	18.4	17.7	12.3	15.0	15.5	10.4	12.9	35.1	22.8	28.9	25.1	14.4	19.6	25.9	14.5	20.1	11.7	7.3	9.5
2006	21.3	13.0	17.1	25.7	13.3	19.1	14.9	10.3	12.6	16.1	8.8	12.4	18.1	10.9	14.5	23.6	14.5	18.9	19.8	13.5	16.6	11.6	7.0	9.2
2007	21.3	12.5	16.8	24.2	13.8	18.7	17.5	10.7	14.0	16.0	8.2	12.0	33.3	22.5	27.8	11.0	7.5	9.2	29.8	17.3	23.3	11.4	7.1	9.2
2008	19.3	11.5	15.4	23.1	13.0	17.8	17.5	10.8	14.1	13.6	9.4	11.5	17.8	12.4	15.1	23.5	14.6	18.9	19.5	13.1	16.2	10.9	6.9	8.9
2009	20.0	11.1	15.5	22.5	12.9	17.4	15.4	105	12.9	14.9	9.0	11.9	17.6	10.1	13.8	26.2	14.1	19.9	19.4	12.5	15.9	10.9	6.7	8.7
2010	20.1	11.4	15.7	21.9	12.1	16.6	15.9	9.0	12.5	14.1	8.8	11.4	17.7	10.1	13.9	22.0	13.0	17.3	38.9	26.0	2.3	10.5	6.4	8.4
2011	19.4	10.9	15.1	20.4	12.2	16.1	14.9	9.2	12.1	14.1	8.9	11.5	15.8	9.1	12.4	23.2	12.4	17.6	20.1	12.1	16.0	10.2	6.3	8.2
2012	17.4	10.5	13.9	19.6	11.3	15.2	13.6	8.1	10.8	12.9	7.4	10.1	14.7	9.4	12.0	20.2	11.5	15.7	19.9	12.1	15.9	9.5	5.8	7.6
2013	16.7	10.3	13.5	19.6	11.2	15.1	14.0	8.4	11.2	12.8	7.4	10.1	6.6	4.4	5.5	21.0	12.2	16.4	18.8	11.1	14.9	9.5	5.8	7.6
2014	16.8	10.0	13.4	18.9	10.3	14.3	12.8	8.0	10.4	12.9	7.1	9.9	16.2	9.9	13.0	19.9	10.6	15.1	17.9	9.5	13.6	9.2	5.4	7.2
2015	17.6	10.1	13.8	18.6	9.5	13.8	12.6	7.4	10.0	12.2	7.3	9.7	14.5	7.9	11.1	19.7	10.8	15.1	19.0	9.7	14.2	9.1	5.1	7.0
2016	18.0	10.5	14.3	18.0	10.0	13.6	13.6	8.6	11.1	11.6	6.3	8.9	16.1	8.9	12.5	20.6	11.5	15.9	16.9	1.7	13.7	9.0	5.4	7.2
2017	17.0	9.5	13.2	15.9	9.0	12.2	12.7	8.1	10.4	10.6	6.4	8.5	14.0	8.1	11.1	19.2	10.7	7.4	16.9	9.7	13.2	8.2	4.9	6.5
2018	15.2	8.5	11.8	15.1	8.4	11.5	12.7	7.4	10.0	12.0	6.1	9.0	5.7	2.8	4.2	19.4	10.0	14.5	16.6	8.8	12.6	24.4	4.6	6.3
2019	16.3	9.0	12.6	13.6	8.2	10.7	13.0	6.9	9.9	9.7	5.9	7.8	14.4	8.0	11.2	19.2	10.1	14.5	15.9	9.3	12.5	7.6	4.5	6.0
2020	13.6	7.7	10.7	13.6	7.1	10.1	13.0	7.1	10.0	9.9	4.7	7.2	12.1	8.2	10.1	16.7	9.0	12.7	16.0	8.5	12.1	7.2	4.1	5.6
2021	14.2	8.6	11.4	14.3	8.3	11.1	13.0	7.3	9.6	10.4	5.4	7.9	14.9	8.2	11.5	18.2	10.6	14.2	16.3	9.2	12.7	7.6	4.6	6.1
2022	17.0	10.0	13.5	15.0	8.5	11.5	7.2	2.8	4.9	10.4	6.0	8.2	7.5	3.3	5.4	19.8	11.0	15.2	18.7	10.2	14.4	8.1	4.8	6.4
2023	13.9	7.5	10.7	14.5	7.8	10.9	5.5	2.4	3.9	9.3	6.0	7.6	5.1	2.2	3.6	17.6	9.9	15.7	16.7	8.8	12.7	7.4	4.2	5.8

RS: Rio Grande do Sul; M: male; FEM: female. Source: Created by the authors.

During the study period, 76,300 deaths from ischemic heart disease were identified in RS. In all macro-regions, higher mortality coefficients were observed among men and in older age groups. The highest mortality levels were found in the South, Central-West, and Metropolitan macro-regions. In RS as a whole, no differences were observed in mortality coefficients from circulatory system diseases between 2020 and 2021. Similarly, no differences were found in mortality levels from ischemic heart diseases during the pandemic period (Table 2).

**Table 2:** Distribution of standardized coefficients of avoidable mortality due to ischemic heart diseases by sex, across health macro-regions and in the state of Rio Grande do Sul, 2000–2023.

Year	Vales			Metropolitan			North			Serra			Missioneira			South			Central West			RS		
	M	FEM	Total	M	FEM	Total	M	FEM	Total	M	FEM	Total	M	FEM	Total	M	FEM	Total	M	FEM	Total	M	FEM	Total
2000	14.1	5.4	9.6	16.6	7.9	11.9	9.8	4.5	7.1	9.9	5.7	7.8	10.1	5.7	7.9	17.2	8.1	9.7	13.2	6.7	9.9	7.5	3.8	5.6
2001	10.7	5.2	7.9	14.0	7.0	10.2	8.5	4.3	6.4	8.6	4.7	6.6	11.6	4.6	8.0	16.9	7.2	7.6	11.9	5.8	8.8	6.6	3.3	5.0
2002	10.9	4.3	7.6	13.4	6.4	9.7	8.2	3.8	6.0	8.5	4.1	6.3	10.3	4.6	7.4	11.3	2.9	8.0	11.6	4.7	8.1	6.1	3.0	4.5
2003	10.1	4.5	7.3	11.7	5.8	8.6	8.7	4.0	6.3	8.1	3.9	6.0	10.0	3.5	6.7	9.1	4.2	8.5	9.7	5.2	7.4	5.7	2.8	4.2
2004	8.3	5.2	6.7	11.0	4.7	7.6	8.5	5.1	6.8	10.2	3.8	6.9	8.7	5.2	6.9	12.9	6.7	4.3	12.8	5.3	9.0	5.7	2.8	4.2
2005	10.3	4.4	7.3	10.2	4.7	7.3	7.1	3.5	5.2	6.6	3.7	5.1	7.7	4.4	6.0	10.9	4.5	8.6	12.2	5.2	8.6	5.1	2.5	3.8
2006	9.2	5.4	7.3	11.6	4.8	8.0	5.9	3.7	4.8	6.8	3.2	5.0	8.9	4.2	6.5	11.0	5.3	7.6	8.5	4.8	6.6	5.1	2.6	3.8
2007	9.6	4.4	7.0	10.4	4.7	7.3	7.3	3.4	5.4	6.9	2.4	4.6	7.2	3.9	5.5	11.1	6.1	6.9	14.4	6.7	10.4	4.8	2.5	3.6
2008	8.4	3.6	5.9	10.3	4.9	7.4	7.5	3.5	5.5	5.0	2.7	3.8	7.8	4.2	6.0	11.2	5.1	6.7	8.1	5.5	6.8	4.7	2.2	3.5
2009	8.2	3.8	6.0	9.6	4.6	6.9	6.8	3.4	5.1	6.3	2.8	4.5	7.3	3.6	5.4	12.6	4.8	6.6	8.8	3.0	5.8	4.6	2.2	3.4
2010	9.6	4.0	6.8	9.5	4.3	6.7	6.2	3.1	4.6	6.6	3.1	4.8	7.6	2.8	5.2	10.2	5.8	6.7	8.5	4.5	6.5	4.5	2.2	3.3
2011	8.0	3.9	6.7	9.4	4.5	6.8	6.2	2.9	4.5	5.3	2.8	4.0	7.0	3.2	5.1	9.9	4.2	6.2	7.9	3.7	5.8	4.1	1.8	3.0
2012	8.0	3.3	5.6	8.6	3.6	5.9	5.0	2.1	3.5	5.1	2.2	3.6	6.8	3.3	5.0	9.3	4.3	6.2	8.4	3.4	5.9	4.2	2.0	3.1
2013	8.2	3.9	6.0	8.6	4.0	6.1	6.0	2.4	4.2	5.3	2.8	4.0	7.3	3.2	5.2	9.5	3.9	3.0	8.1	4.0	6.0	4.1	1.9	3.0
2014	8.1	4.1	6.1	8.3	3.6	5.8	5.5	2.8	4.1	5.5	2.3	3.9	6.9	3.7	5.3	9.8	3.9	5.6	8.0	2.6	5.3	4.0	1.9	2.9
2015	8.3	4.5	6.4	8.4	3.5	5.8	5.4	2.6	4.0	5.1	2.0	3.5	5.9	2.9	4.4	8.2	4.3	5.6	8.6	3.0	5.7	3.9	1.8	2.8
2016	9.2	4.4	6.8	7.6	3.4	5.3	6.5	2.9	4.6	4.3	1.7	2.9	7.1	2.9	4.9	8.6	3.9	5.0	7.9	3.3	5.5	3.5	1.7	2.6
2017	7.8	4.0	5.9	6.6	3.1	4.7	5.4	2.6	4.0	4.7	2.0	3.3	6.6	3.0	4.8	7.2	4.0	3.0	8.0	3.6	5.7	3.4	1.5	2.5
2018	7.4	3.1	5.2	6.0	2.7	4.2	6.1	2.7	4.4	5.3	1.7	3.5	6.9	3.0	4.9	8.4	3.0	5.6	6.8	2.8	4.8	3.4	1.5	2.5
2019	8.7	3.4	6.0	5.4	2.6	3.9	6.0	2.6	4.3	4.2	2.1	3.1	6.9	2.9	4.8	8.0	3.4	5.6	7.4	3.2	5.2	3.3	1.6	2.4
2020	6.8	3.1	4.9	5.3	2.1	3.6	5.8	2.3	4.0	4.6	1.6	3.1	5.6	3.0	4.3	7.2	3.0	5.0	6.2	2.5	4.3	3.0	1.3	2.2
2021	6.7	3.2	4.9	5.4	2.5	3.9	5.0	2.3	3.6	4.3	2.0	3.1	5.9	2.3	4.1	8.6	4.4	6.4	6.2	2.4	4.2	3.1	1.5	2.3
2022	7.7	2.8	5.2	6.0	2.9	4.4	7.2	2.8	4.9	4.5	1.9	3.2	7.5	3.3	5.4	8.9	3.6	6.2	8.0	3.0	5.4	3.4	1.5	2.4
2023	6.6	2.2	4.4	6.5	2.5	4.4	5.5	2.4	3.9	3.8	1.7	2.7	5.1	2.2	3.6	7.8	3.7	5.7	6.8	3.0	4.9	3.1	1.3	2. 2

RS: Rio Grande do Sull; M: male; FEM: female. Source: Created by the authors.

The trend analysis of avoidable mortality from both circulatory system diseases and ischemic heart diseases showed a reduction across all macro-regions and in the state overall. The Missions macro-region showed the greatest decline, with a reduction of 2.26 deaths per 10,000 population per year (Table 3).

**Table 3:** Trend analysis of avoidable mortality rates per 100,000 inhabitants from circulatory system diseases and ischemic heart diseases according to sex in the health macro-regions and in the State of Rio Grande do Sul, 2000 to 2023.

	Circulatory system diseases			Ischemic heart diseases		
	Coefficient	95%CI	Value of p	Coefficient	95%CI	Value of p
Central West						
Male	-0.58	-0.79; -0.37	<0.001	-0.02	-0.35; 0.32	0.925
Feminine	-0.40	-0.57; -0.23	<0.001	-0.04	-0.20; 0.10	0.511
Total	-0.48	-0.67; -0.31	<0.001	-0.01	-0.28; 0.26	0.941
Metropolitan						
Male	-0.72	-0.91; -0.65	<0.001	-0.40	-0.50; -0.31	<0.001
Feminine	-0.46	-0.54; -0.37	<0.001	-0.21	-0.26; -0.15	<0.001
Total	-0.61	-0.73; -0.50	<0.001	-0.30	-0.38; -0.22	<0.001
Missioneira						
Male	-0.83	-1.31; -0.35	0.002	-0.19	-0.26; -0.13	<0.001
Feminine	-0.56	-0.86; -0.26	0.001	-0.10	-0.13; -0.08	<0.001
Total	-0.68	-0.89; -0.47	<0.001	-0.15	-0.19; -0.11	<0.001
North						
Male	-0.61	-0.78; -0.44	<0.001	-0.14	-0.21; -0.08	<0.001
Feminine	-0.39	-0.48; -0.30	<0.001	-0.09	-0.13; -0.06	<0.001
Total	-0.50	-0.62; -0.38	<0.001	-0.12	-0.17; -0.07	<0.001
Serra						
Male	-0.50	-0.59; -0.41	<0.001	-0.23	-0.29; -0.17	<0.001
Feminine	-0.35	-0.44; -0.26	<0.001	-0.15	-0.20; -0.10	<0.001
Total	-0.40	-0.53; -0.27	<0.001	-0.19	-0.24; -0.14	<0.001
South						
Male	-0.49	-0.75; -0.24	0.01	-0.30	-0.43; -0.18	<0.001
Feminine	-0.24	-0.38; -0.09	0.02	-0.12	-0.19; -0.06	<0.001
Total	-0.42	-0.75; -0.09	0.016	-0.26	-0.37; -0.15	<0.001
Vales						
Male	0.62	-0.71; 1.96	0.340	-0.18	-0.25; -0.12	<0.001
Feminine	-0.25	-0.36; -0.14	<0.001	-0.10	-0.14; -0.06	<0.001
Total	-0.54	-0.63; -0.46	<0.001	-0.16	-0.22; -0.11	<0.001
RS						
Male	2.00	-15.7; 19.7	0.817	1.73	-3.11; 6.58	0.467
Feminine	-0.22	-0.26; -0.18	<0.001	-0.09	-0.11; -0.07	<0.001
Total	-0.28	-0.34; -0.23	<0.001	-0.13	-0.16; -0.10	<0.001

RS: Rio Grande do Sul; CI: confidence interval. Source: Created by the authors

Regarding the trend analysis by sex, a decline in mortality from circulatory system diseases was observed among women in all macro-regions and in RS. Among men, most macro-regions also showed a decreasing trend, except for the Vales macro-region and the state overall, where the results were stable. In relation to ischemic heart diseases, a reduction in mortality was observed in both sexes across most macro-regions, except in the Central-West and among men in RS as a whole (Table 3).

Trend analysis further indicated a reduction in mortality in RS from circulatory system diseases and ischemic heart diseases across both sexes and all age groups (Supplementary Material).

## DISCUSSION

The present study showed a reduction in mortality from circulatory system diseases and ischemic heart diseases among women in almost all health macro-regions, except for the Central-West. Among men, a decrease in mortality trends for circulatory system diseases was also observed, except in the Vales macro-region and in the state overall. The analysis also demonstrated higher mortality among men and a progressive increase with age.

The reduction in mortality follows the global trend, and the predominance of mortality among men and older adults has also been reported in Brazilian studies^12,13,14^. However, an ecological study conducted in the United States revealed a reversal in the declining trend of mortality from circulatory system diseases during the pandemic period, with an estimated 228,000 excess deaths in 2022^15^. Rosa et al., in an ecological study conducted in the health regions and capital of Rio de Janeiro between 1996 and 2016, found stability or an increase in ischemic heart disease mortality in at least 50% of localities^16^.

Despite the observed reduction, circulatory system diseases and ischemic heart diseases remain the leading causes of death worldwide^4^, in Brazil^17^, and in RS across all age groups.

The reduction observed is paradoxical considering population aging, as data from the 2022 Census showed that RS had the highest proportion of individuals aged 65 years or older in Brazil (14.1%). However, several health policies and actions were implemented in Brazil during the study period, which may help explain the decrease in mortality^18^.

Initially, the maturation of the Unified Health System ensured the universality of healthcare, establishing a vast network of primary health care through the expansion of the Family Health Strategy^19^. The Reorganization Plan for the Care of Hypertension and Diabetes Mellitus was also launched, aimed at case identification, staff training, diagnosis, and medication distribution^20^. During this period, there was also an expansion of medications provided by the Popular Pharmacy Program, including the availability of drugs for dyslipidemia control. Moreover, the period was marked by the success of anti-smoking measures^21^ and the promotion of healthy lifestyle habits through the Strategic Action Plan to Tackle Noncommunicable Chronic Diseases^22^, all of which constitute a set of policies that may have contributed to the observed decline in mortality.

Ecological studies have inherent limitations in providing evidence due to their study design characteristics and their susceptibility to reporting and classification errors when using secondary data. Mortality data were found to be underestimated due to delays in conducting the census. This study used the population estimates available from DATASUS for Rio Grande do Sul and its macro-regions up to 2021 to calculate mortality coefficients; however, these estimates were outdated compared to actual figures. According to the 2022 Census, the actual population of RS was 10,882,965 inhabitants, whereas the DATASUS projection for 2021 was 11,466,630 inhabitants, representing an overestimation.

Additionally, this study did not incorporate variables that could explain differences between macro-regions, such as socioeconomic factors or differences related to the complexity of health systems^23^. Nevertheless, the Mortality Information System is the most established health information system in Brazil, and a recent review confirmed its improvement and qualification in the states of the South Region^24^. Direct standardization eliminated the effects of differences in age structure between macro-regions.

Ecological studies are rapid, inexpensive, and straightforward to conduct, offering the potential to provide an overview of the health situation. They can be useful for health managers, and it is recommended that transcendent diseases be continuously monitored^10,25^.

## CONCLUSION

The findings of the present study highlighted a reduction in mortality from circulatory system diseases and ischemic heart diseases, despite the continuous increase in the elderly population resulting from the inversion of the age pyramid. The observed decline in mortality rates follows global trends and is consistent with the results of other Brazilian studies regarding the predominance of mortality among men and older adults.

It is recommended that municipalities monitor the main causes of avoidable mortality to improve health policies and actions, given that such information is available and can be analyzed simply and quickly.

## Data Availability

The datasets generated and/or analyzed during the current study are available from the corresponding author upon reasonable request.
